# Estimated Visceral Adipose Tissue, but Not Body Mass Index, Is Associated with Reductions in Glomerular Filtration Rate Based on Cystatin C in the Early Stages of Chronic Kidney Disease

**DOI:** 10.1155/2014/574267

**Published:** 2014-05-04

**Authors:** Ana Karina Teixeira da Cunha França, Alcione Miranda dos Santos, João Victor Salgado, Elane Viana Hortegal, Antônio Augusto Moura da Silva, Natalino Salgado Filho

**Affiliations:** ^1^Departamento of Health Sciences, Federal University of Maranhão, São Luís, Brazil; ^2^Collective Health Postgraduate Program, Federal University of Maranhão, São Luís, Brazil; ^3^Clinical Chemistry Service, Kidney Disease Prevention Centre, University Hospital, Federal University of Maranhão, São Luís, Brazil; ^4^Kidney Disease Prevention Centre, University Hospital, Federal University of Maranhão, São Luís, Brazil; ^5^Department of Medicine I, Federal University of Maranhão, São Luís, Brazil

## Abstract

Information on the association between obesity and initial phases of chronic kidney disease (CKD) is still limited, principally those regarding the influence of visceral adipose tissue. We investigated whether the visceral adipose tissue is more associated with reductions in glomerular filtration rate (GFR) than total and abdominal obesity in hypertensive individuals with stage 1-2 CKD. A cross-sectional study was implemented which involved 241 hypertensive patients undergoing treatment at a primary health care facility. GFR was estimated using equations based on creatinine and cystatin C levels. Explanatory variables included body mass index (BMI), waist circumference (WC), and estimated visceral adipose tissue (eVAT). The mean age was 59.6 ± 9.2 years old and 75.9% were female. According to BMI, 28.2% of subjects were obese. Prevalence of increased WC and eVAT was 63.9% and 58.5%, respectively. Results from the assessment of GFR by BMI, WC, and eVAT categories showed that only women with increased eVAT (≥150 cm^2^) had a lower mean GFR by Larsson (*P* = 0.016), Levey 2 (*P* = 0.005), and Levey 3 (*P* = 0.008) equations. The same result was not observed when the MDRD equation was employed. No association was found between BMI, WC, eVAT, and GFR using only serum creatinine. In the early stages of CKD, increased eVAT in hypertensive women was associated with decreased GFR based on cystatin C.

## 1. Introduction

Chronic kidney disease (CKD) has been assuming global importance because of the exponential increase in the number of cases registered in the last few decades. The CKD epidemic calls for public policies targeting prevention, early diagnosis, and intervention for this disease [1].

The global obesity epidemic translates into markedly heightened CKD prevalence [2, 3]. The public health obesity crisis is expected to encompass 700 million adults by 2015 [4]. Aside from being a major risk factor for the development of diabetes and hypertension, the principal causes of CKD in the western world obesity may have adverse effects on kidney function independently of such intermediate disease states [5, 6].

Conversely, the role of body-fat distribution in the development of CKD is less clear. The progression of renal failure is associated with a spontaneous decrease in dietary protein intake and consequent weight loss. Thus, most nutritional indices in CKD patients worsen as CrCl decreases [7], which compromises the assessment of the association between obesity and glomerular filtration rate in patients at advanced stages of the disease.

In addition, the changes in physiological functions that accompany obesity depend to a certain extent on the regional adipose tissue distribution [8]. Studies have shown that total obesity assessed by the body mass index (BMI) is associated with the development of CKD [6, 9], whereas other studies have found that anthropometric indexes of abdominal obesity, waist circumference (WC), and waist-to-hip ratio (WHR) are more sensitive predictors of CKD [8, 10]. Although such anthropometric measurements have good correlation with abdominal adiposity, they do not differentiate the visceral from subcutaneous adiposities. Moreover, the effects of the visceral adipose tissue cannot be completely explained by BMI and WC [11]. Interestingly, visceral adipose tissue (VAT) has shown stronger association with the most metabolic risk factors when compared to subcutaneous abdominal tissue [12]. However, there are few studies using this index to evaluate the correlation between obesity and CKD [13, 14].

The most sensible methods to measure VAT are computed tomography (CT) and magnetic resonance imaging (MRI) [15]. Nevertheless, these procedures are inadequate to screen large populations, since they are time consuming, expensive, and specialized equipment that may expose the individuals to ionizing radiation [16, 17]. To circumvent such limitations, predictive equations were validated to estimate VAT from simple anthropometric measures [16–18]. In this setting, these predictive equations can be considered as simple, cost effective, and easy measurement tools to assess VAT and surrogate the imaging techniques [16].

The glomerular filtration rate (GFR) is an effective indicator of kidney function and creatinine-based estimating equations are recommended in clinical practice [8]. Yet, cystatin C has shown to be as good as or even better as a predictor of GFR [19, 20] because it is independent of muscle mass [20, 21] and it is less prone to being influenced by body composition [4]. On the other hand, some studies have reported an association between obesity and elevated serum cystatin C levels [14, 22, 23]. However, the contribution of adipose tissue in this association is not clear.

The majority of studies highlight the association between total and/or abdominal obesity with well-defined CKD (GFR < 60 mL/min/1.73 m^2^). Information on the association between obesity and initial phases of CKD is limited, mainly those regarding the influence of visceral adipose tissue. Therefore, the objective of the present study was to investigate whether the estimated visceral adipose tissue is more associated with reductions in glomerular filtration rate (GFR) than body mass index (BMI) and waist circumference (WC) in hypertensive individuals with stage 1-2 CKD.

## 2. Methods

A cross-sectional study involving hypertensive patients was performed from January to June 2008. The subjects were enrolled at the HiperDia Program of the Ministry of Health and received regular follow-ups in a basic health unit in São Luis, Brazil.

The calculus of the size of the sample was performed considering the population of 675 hypertensive patients enrolled at the HiperDia Program of the Vila Embratel Health Center. To calculate the sample, the expected prevalence of GFR < 60 mL/min/1.73 m^2^ 22% [19] in hypertensive patients, error margin of 4.5%, and confidence level of 95% were considered. The total number of hypertensive patients considered was 220 and, considering possible losses during the research, the size of sample was increased by 20%, totaling 264 patients.

The initial participant selection was obtained by using a list of names of hypertensive patients enrolled at the HiperDia Program from a selected basic health unit. The study design included a random sample, without replacement.

We included individuals from both sexes, aged ≥40, diagnosed with hypertension enrolled at Program Hiperdia, and regularly monitored at a selected basic health unit. We did not include patients who are pregnant, patients who have eGFR < 60 mL/min/1.73/m^2^, severe cardiac insufficiency, malignancy or infection, and thyroid dysfunction, and patients who use glucocorticoids. There were 241 patients in the completed sample.

The adoption of these exclusion criteria is justified by the following: (1) thyroid dysfunction or use of glucocorticoids that may interfere with eGFR based on cystatin C; (2) the eGFR < 60 mL/min/1.73/m^2^ to exclude subjects with moderate to advanced CKD; and (3) the age <40 years old to exclude younger individuals, whose main causes of CKD are infectious diseases.

Fasting blood samples and anthropometrical data were collected and a clinical interview on sociodemographic and clinical data was performed. Blood samples were sent immediately to the Clinical Chemistry Laboratory at the University Hospital and processed on the same day. Plasma glucose, triglycerides, total cholesterol, high-density lipoprotein-cholesterol levels, and serum creatinine levels (Jaffe's kinetic assay) were obtained using the ADVIA 1650 by the immunoturbidimetric method using reagents, controls, and calibrators from Bayer. Microalbuminuria was determined by ELISA using a 24 hr urine collection.

Renal function was assessed by eGFR based on both serum creatinine and cystatin C levels. Cystatin C was measured using a BNA nephelometer, which utilizes a particle-enhanced immunonephelometric assay (*N Latex Cystatin C, Behring Nephelometer 100 system analyzer, Marburg, Germany*). The equations to estimate GFR using serum creatinine and cystatin C levels are described below. According to K/DIGO (Kidney Disease: Improving Global Outcome) recommendations, the simplified modification of diet in renal disease (MDRD) equation was used as a reference to screen CKD [24]. The “African-American” constant was not included in the calculation.

*Simplified MDRD Equation [24].* eGFR (mL/min/1.73 m^2^) = 186.3 × creatinine (mg/dL)^−1.154^  × age (yrs)^−0.203^  × 0.742 if female and × 1.210 if African American.

*Formula 1 (Larsson et al. [25]).* eGFR (mL/min/1.73 m^2^) = 77.239 × cystatin C (mg/L)^−1.2623^.

*Formula 2 (Levey et al.* *[26]).* eGFR (mL/min/1.73 m^2^) = 127.7 × cystatin C (mg/L)^−1.17^  × age (yrs)^−0.13^ if male and × 0.91 if female.

*Formula 3 (Levey et al.* *[26]).* eGFR (mL/min/1.73 m^2^) = 177.6 × creatinine (mg/dL)^−0.65^ × cystatin C (mg/L)^−0.57^  × age (yrs)^−0.20^ if male and × 0.82 if female.

Blood pressure was measured using a digital sphygmomanometer (Omron) by an indirect method with the patient resting in the seated position. Anthropometric parameters included body weight, height, and WC. BMI was calculated by the ratio of weight (kg)/height (m^2^), and the patients were classified as nonobese if BMI < 30.0 kg/m^2^ and obese if BMI ≥ 30.0 kg/m^2^. Abdominal obesity was assessed by WC, measured at the midpoint between the lowest rib and the iliac crest. WC was classified as very high risk if ≥88 cm in women and ≥102 cm in men. Estimated visceral adipose tissue (eVAT) was assessed by the predictive equation −453.7 + (6.37 × WC) if male and −370.5 + (4.04 × WC)+(2.62 × age) if female, as proposed by Bonora et al. [17] eVAT was classified as very high if ≥150 cm^2^ [18].

Since our sample comprised hypertensive and middle age patients, we aimed to evaluate the effect of global, abdominal and visceral obesities on renal function. We considered the higher cutoffs of the nutritional indices.

Statistical analyses were performed using the Stata program (version 10.0). Differences in means between groups were assessed using Student's *t*-test or Mann-Whitney* U* test. Normality of quantitative variables was analyzed by Shapiro Wilk test. The level of significance was 5%. As the distribution of estimated GFR by creatinine and cystatin C based equations was not normal, the Wilcoxon test was used to evaluate the difference between eGFR by MDRD and the other equations.

The study was approved by the Research Ethics Committee of the Federal University of Maranhão, Brazil (Protocol 1977/2007). Patients who agreed to participate gave written informed consent.

## 3. Results

A total of 241 patients were assessed. Mean age was 59.6 ± 9.2 years old and 75.9% were females. According to BMI, 28.2% of patients were obese. The prevalence of abdominal and visceral obesity assessed by WC and eVAT was 63.9% and 58.5%, respectively. The prevalence of diabetes was 31.1% and no statistical differences in demographic, nutritional, and kidney function variables were observed between diabetic and nondiabetic hypertensive patients (data not presented in the table).

Table 1 shows the clinical, biological, and anthropometric characteristics of the patients by gender. Women showed higher serum total cholesterol levels than men (214.2 ± 44.2 mg/dL versus 191.9 ± 40.5 mg/dL, *P* < 0.001) whereas men demonstrated higher mean levels of serum creatinine (1.0 ± 0.1 mg/dL versus 0.7 ± 0.1 mg/dL, *P* < 0.001) and serum cystatin C (0.91 ± 0.18 mg/dL versus 0.82 ± 0.15 mg/dL, *P* < 0.001) than women. When the other variables were compared by gender, no significant differences were observed.

In comparative analysis between eGFR based on cystatin C equations (Larsson, Levey 2, and Levey 3) and eGFR by MDRD equation, only the eGFR by Larsson equation showed statistically significant difference (100.3 ± 23.3 mL/min/1.73 m^2^ versus 89.6 ± 19.5 mL/min/1.73 m^2^, *P* < 0.001). Moreover, when the eGFR by the four equations was compared by gender, males presented a lower mean of GFR estimated by Larsson formula than females (91.1 ± 20.5 mg/dL versus 103.2 ± 23.4 mg/dL, *P* < 0.001) (Table 2).

Results from the assessment of GFR by BMI, WC, and eVAT categories highlighted that women with increased eVAT (≥150 cm^2^) had a lower eGFR mean by the Larsson (99.5 ± 21.7 cm^2^ versus 109.2 ± 24.9 cm^2^, *P* = 0.016), Levey 2 (86.1 ± 18.1 cm² versus 95.4 ± 20.6 cm^2^, *P* = 0.005), and Levey 3 (86.8 ± 14.8 cm^2^ versus 94.8 ± 18.6 cm^2^, *P* = 0.008) equations, in comparison with women with normal eVAT (<150 cm²) (Table 3).

## 4. Discussion

In this study, the mean eGFR using several equations based on serum cystatin C (Larsson, Levey 2, and Levey 3) was significantly lower in women with higher eVAT (≥150 cm^2^) compared with those with normal eVAT. Moreover, both men and women who were classified as obese by their BMI, very high WC, and eVAT did not show differences in eGFR by the MDRD equation, when compared with patients in the group with normal categories for these indices. Furthermore, we only found significant differences between mean eGFR by the MDRD from the Larsson equations. This difference may be explained by the absence of the variables gender, age, and serum creatinine in the Larsson formula.

In the current study, there are no differences between the mean eGFR by the MDRD equation and all nutritional indices that might be attributed to the inclusion of serum creatinine in this equation [24]. Creatinine is considerably affected by the amount of lean mass [21] since it is generally formed in the muscle [27]. Thus, the MDRD equation might not be useful to detect GFR alterations in individuals with reduced lean mass, such as the elderly, undernourished people [27], and possibly obese individuals.

Serum cystatin C is an endogenous, 13-kilodalton protein filtered by the glomeruli and reabsorbed and catabolized by the tubular epithelial cells with only small amounts excreted in the urine, and it is reported to be generated at a relatively constant rate [28]. Thus, it was anticipated that cystatin C would provide a better estimate of GFR than estimating equations based on serum creatinine [26], because its concentration depends directly on the GFR [28] and it is independent of muscle mass [21]. It also seems to be a better indicator of GFR in obese patients because it is less prone to being influenced by body composition [4, 29]. Besides, cystatin C is known to be a sensitive marker of small reductions in renal function and has been shown to detect “preclinical kidney disease,” which is associated with increased risk of CKD progression and cardiovascular death [28, 30].

On the other hand, studies have shown that cystatin C levels may be dependent of adipose tissue [9, 22], increasing directly as BMI increases, and, thus, it can underestimate GFR in individuals with higher levels of obesity [31, 32]. In the present study, the obese subgroup presented mean BMI values that were representative of mild obesity and cystatin C levels were similar in obese and nonobese groups in both men (*P* = 0.104) and women (*P* = 0.484). Similarly, Schück et al. [33] and Friedman et al. [4] found no association between serum cystatin C levels and BMI in individuals classified with moderate or severe obesity.

In another study, Sledzinski et al. [34] tested serum cystatin C levels in 27 obese patients before bariatric surgery and 6 months postoperatively. The authors observed that changes in body and fat mass after surgery did not contribute directly to differences in changes of serum cystatin C concentrations. This study also suggests that obesity contributes to the development of CKD, which in turn contributes to the increase in serum cystatin C levels in obese patients [29, 34]. Interestingly, Marwyne et al. [35], using a direct measurement of GFR, demonstrated that cystatin C-based eGFR equations were more accurate, sensitive, and specific in overweight and obese subjects compared to creatinine-based eGFR equations.

It is necessary to acknowledge that the diagnostic accuracy of BMI to diagnose obesity is restricted. BMI has clear limitations to differentiate adipose tissue from lean mass in intermediate BMI ranges. Furthermore, physiological alterations that resulted from obesity depend to a certain degree on the regional distribution of adipose tissue [8]. Visceral adiposity, more than increased BMI and subcutaneous adiposity, has been associated with the deterioration of renal function and metabolic risk [13].

In this context, visceral obesity is related to increased levels of blood pressure, insulin resistance, and dyslipidemia [36]. Visceral obesity associated with insulin resistance leads not only to compensatory hyperinsulinemia but also to inadequate activation of the renin-angiotensin system and oxidative stress to the kidney. This can lead to increased blood pressure [37], salt-sensitive blood pressure, excess of aldosterone, glomerular hypertension, endothelial dysfunction, and vasoconstriction [8]. In addition, visceral adiposity can physically compress the kidneys, increasing the intrarenal pressure and tubular reabsorption. The consequences of the renal injury are continuous loss of GFR, increases in blood pressure, and worsening cardiovascular morbidity and mortality [38].

In a study conducted by Young et al. [14], they found evidence that VAT is associated with elevated cystatin C levels, supporting the role of visceral fat in reducing the GFR. This is in accordance with our findings, as we found an association between eVAT ≥ 150 cm² and lower mean eGFR by three cystatin C based equations in women. However, this association was not observed in males. One possible reason for this finding could be due to the small size of the sample, although the male gender was represented in this population. Additionally, our results were supported by other recent studies which reported that both visceral and subcutaneous adiposity were associated with kidney disease, as defined by only cystatin C based equations, but not when defined using the creatinine based equations [13].

This study has certain weaknesses. Due to the study design, a causal association between GFR and visceral obesity in hypertensive subjects was not possible to be established. Since the sample was composed of hypertensive patients in stage 1-2 CKD, mostly women from primary health care, these results cannot be generalized to other groups. Although a gold-standard method was not used to assess GFR and VAT, this study has certain strengths. Firstly, GFR was estimated by serum cystatin C based equations, which is still not widely used in routine clinical practice, mainly in public health care. Although cystatin C has clear superiority over serum creatinine and already has a standardized assay, its high cost is still a problem [39]. Nowadays, in our opinion, the best equation to be applied to this study would be the CKD-EPI equation using the new and calibrated cystatin C. However, our study was developed at basic health care with limited access to standardized cystatin C. Secondly, despite VAT being estimated indirectly by the equation, our findings do not differ from those observed in the literature that evaluated the association between cystatin C and VAT measured by computed tomography [14]. Thus, eVAT could be used as a simple and low cost alternative to assess VAT at primary health care.

In summary, the mean values of eGFR based on cystatin C equations (Levey 2 and Levey 3) were similar to the mean values obtained by the MDRD equation. Only in hypertensive women, the eGFR by the Larsson equation showed significant difference in relation to the eGFR by the MDRD equation. In this study, BMI and WC were not associated with eGFR based on serum creatinine and cystatin C. Only increased VAT in hypertensive women was associated with reductions in eGFR values by cystatin C equations. The present findings demonstrate the importance of using eVAT to control the obesity in hypertensive patients with initial stages of CKD and consequently preventing CKD progression and cardiometabolic disorders.

## Figures and Tables

**Table 1:** Clinical, biological, and anthropometric characteristics of the study participants by gender.

Variables	Total *n* = 241	Male *n* = 58	Female *n* = 183	*P* value
Age (yrs)	59.6 ± 9.2	61.5 ± 9.5	59.0 ± 9.0	0.093
Fasting glucose (mg/dL)	101.8 ± 57.8	89.4 ± 36.5	105.8 ± 62.7	0.124
Cholesterol (mg/dL)	208.1 ± 44.3	191.9 ± 40.5	214.2 ± 44.2	<0.001
HDL-cholesterol (mg/dL)	46.1 ± 11.6	43.5 ± 9.7	47.0 ± 12.0	0.074
Triglycerides (mg/dL)	146.9 ± 75.5	137.4 ± 63.7	149.8 ± 78.8	0.470
Microalbuminuria (mg/24 h)	30.4 ± 58.5	41.6 ± 67.8	26.8 ± 55.0	0.336
SBP (mmHg)	146.5 ± 19.1	150.2 ± 18.2	145.4 ± 19.3	0.126
DBP (mmHg)	87.9 ± 10.7	88.7 ± 10.2	87.6 ± 10.9	0.693
Duration of hypertension (yrs)	7.6 ± 6.6	7.2 ± 6.3	7.7 ± 6.6	0.695
Serum creatinine (mg/dL)	0.81 ± 0.16	1.0 ± 0.1	0.7 ± 0.1	<0.001
Serum cystatin C (mg/L)	0.84 ± 0.16	0.91 ± 0.18	0.82 ± 0.15	<0.001
BMI (kg/m^2^)	27.8 ± 4.6	26.6 ± 3.7	28.1 ± 4.8	0.058
WC (cm)	95.3 ± 11.3	95.5 ± 10.2	95.2 ± 11.7	0.630
VAT (cm^2^)	165.5 ± 55.9	154.7 ± 65.2	168.9 ± 52.4	0.166

HDL: high density lipoprotein cholesterol; SBP: systolic blood pressure; DBP: diastolic blood pressure; BMI: body mass index; WC: waist circumference; eVAT: estimated visceral adipose tissue. Data are expressed as mean ± SD.

**Table 2:** Differences between the glomerular filtration rate estimated by the modification of diet in renal disease and glomerular filtration rate estimated by Larsson, Levey 2, and Levey 3 equations by gender.

Variables	Total *n* = 241	Male *n* = 58	Female *n* = 183	*P* value
GFR MDRD (mL/min/1.73 m^2^)	89.6 ± 19.5	89.5 ± 61.2	89.6 ± 20.1	0.780
GFR Larsson (mL/min/1.73 m^2^)	100.3 ± 23.3	91.1 ± 20.5	103.2 ± 23.4	<0.001
Dif.	−10.7 ± 22.9	−1.6 ± 18.2	−13.6 ± 23.4	
*P* value	<0.001	0.503	<0.001	
GFR Levey 2 (mL/min/1.73 m^2^)	89.1 ± 19.5	87.3 ± 19.1	89.6 ± 19.6	0.608
Dif.	0.5 ± 20.2	2.2 ± 17.6	−0.0 ± 21.0	
*P* value	0.685	0.379	0.963	
GFR Levey 3 (mL/min/1.73 m^2^)	89.1 ± 16.7	86.7 ± 16.6	89.9 ± 16.8	0.265
Dif.	0.5 ± 11.7	2.8 ± 10.7	−0.2 ± 11.9	
*P* value	0.683	0.172	0.206	

GFR: glomerular filtration rate (mL/min/1.73 m²); MDRD: modification of diet in renal disease equation; Larsson: equation with cystatin C and without age and gender adjustment or based only on cystatin C; Levey 2: equation with cystatin C and age and gender adjustment; Levey 3: equation with cystatin C, creatinine, age, and gender adjustment; Dif.: difference between the GFR estimated by MDRD and the other equations. Data are expressed as mean ± SD.

**Table 3:** Glomerular filtration rate estimated by the modification of diet in renal disease, Larsson, Levey 2, and Levey 3 equations by categories of body mass index, waist circumference, and visceral adipose tissue.

Variables	GFR (mL/min/1.73 m^2^)								
	BMI		*P* value	WC		*P* value	VAT		*P* value
	Nonobese	Obese		Normal	High		Normal	High	
Male	*n* = 44	*n* = 14		*n* = 39	*n* = 19		*n* = 30	*n* = 28	
MDRD	89.5 ± 19.3	89.7 ± 12.9	0.842	90.0 ± 19.2	88.7 ± 15.0	0.993	90.2 ± 19.8	88.8 ± 15.8	0.963
Larsson	88.3 ± 19.9	99.8 ± 20.7	0.104	88.2 ± 20.3	96.9 ± 20.4	0.161	89.7 ± 19.9	93.6 ± 21.5	0.559
Levey 2	84.5 ± 18.6	95.9 ± 18.9	0.064	84.8 ± 18.9	92.4 ± 19.0	0.205	85.9 ± 18.5	88.7 ± 20.0	0.554
Levey 3	85.4 ± 17.6	90.9 ± 12.4	0.217	85.8 ± 17.8	88.5 ± 14.1	0.534	86.3 ± 17.7	87.2 ± 15.5	0.618
Female	*n* = 129	*n* = 54		*n* = 48	*n* = 135		*n* = 70	*n* = 113	
MDRD	90.3 ± 21.0	88.1 ± 17.8	0.738	91.7 ± 23.7	88.9 ± 18.7	0.670	93.4 ± 23.7	87.3 ± 17.2	0.122
Larsson	103.7 ± 23.2	102.1 ± 24.0	0.484	106.7 ± 23.2	102.0 ± 22.4	0.251	109.2 ± 24.9	99.5 ± 21.7	0.016
Levey 2	89.8 ± 19.3	89.3 ± 20.5	0.643	92.3 ± 18.7	88.7 ± 19.8	0.284	95.4 ± 20.6	86.1 ± 18.1	0.005
Levey 3	89.9 ± 16.4	89.7 ± 17.9	0.932	92.2 ± 18.1	89.0 ± 16.3	0.382	94.8 ± 18.6	86.8 ± 14.8	0.008

GFR: glomerular filtration rate (mL/min/1.73 m^2^); BMI: body mass index was classified as nonobese if <30.0 kg/m² and obese if ≥30.0 kg/m²; WC: waist circumference was assessed as high if ≥88 cm in women and ≥102 cm in men; eVAT: estimated visceral adipose tissue was defined as high if greater than ≥150 cm² for men and women; MDRD: modification of diet in renal disease equation; Larsson: equation with cystatin C and without age and gender adjustment or based only on cystatin C; Levey 2: equation with cystatin C and age and gender adjustment; Levey 3: equation with cystatin C, creatinine, age, and gender adjustment; Dif.: difference between the GFR estimated by MDRD and the other equations. Data are expressed as mean ± SD.
